# SERCA2a Protein Levels Are Unaltered in Human Heart Failure

**DOI:** 10.1161/CIRCULATIONAHA.123.064513

**Published:** 2023-08-14

**Authors:** Isabella Ragone, Javier Barallobre-Barreiro, Kaloyan Takov, Konstantinos Theofilatos, Xiaoke Yin, Lukas Emanuel Schmidt, Nieves Domenech, Maria Generosa Crespo-Leiro, Stephanie M. van der Voorn, Aryan Vink, Toon A.B. van Veen, Csaba Bödör, Béla Merkely, Tamás Radovits, Manuel Mayr

**Affiliations:** King’s British Heart Foundation Centre, King’s College London, UK (I.R., J.B.-B., K.T., K.T., X.Y., M.M.).; Department of Internal Medicine II, Division of Cardiology, Medical University of Vienna, Austria (L.E.S., M.M.).; Instituto de Investigación Biomédica de A Coruña (INIBIC)-CIBERCV, Complexo Hospitalario Universitario de A Coruña (CHUAC), Universidade da Coruña, Spain (N.D., M.G.C.-L.).; Departments of Medical Physiology (S.M.v.d.V., T.A.B.v.V.), University Medical Centre Utrecht, the Netherlands.; Division of Heart & Lungs and Pathology (A.V.)., University Medical Centre Utrecht, the Netherlands.; Department of Pathology and Experimental Cancer Research (C.B.), Semmelweis University, Budapest, Hungary.; Heart and Vascular Center, Department of Cardiology (B.M., T.R.), Semmelweis University, Budapest, Hungary.

Sarcoplasmic reticulum Ca^2+^ATPase 2a (SERCA2a) and its main regulator, phospholamban (PLN), affect Ca^2+^ handling in cardiac muscle and have been implicated in heart failure (HF). Studies on patients with HF have reported reduced SERCA2a and PLN transcript levels, but findings at the protein level have been less consistent.^1,2^ The prior studies have been limited by small numbers of patients with heterogeneous causes of HF.^1,2^ Despite promising results in animal models, clinical trials of gene therapy for SERCA2a overexpression have not yet shown significant improvement in outcomes.^3^ Therefore, our aim was to conduct a comprehensive investigation of the protein levels of SERCA2a and PLN in patients with HF.

In this study, we evaluated the transcript and protein levels of SERCA2a and PLN in cardiac explants obtained from 3 groups: 114 patients with HF with dilated cardiomyopathy (DCM; 77% male; age, 51.4±11.4 years), 65 patients with HF with ischemic heart disease (IHD; 85% male; age, 57.6±6.5 years), and 57 patients with nonfailing hearts (35% male; age, 67.8±9.3 years) as controls (Figure [A]). Human left ventricles were obtained from A Coruña University Hospital’s Advanced Heart Failure and Heart Transplantation Unit (Spain) and the Transplantation Biobank of the Heart and Vascular Center at Semmelweis University (Hungary). Written consent and institutional approval were obtained (REC LRS-17/18-5080, Entry-17440; REC Entry 2015/312; ETT TUKEB 7891/2012/EKU [119/PI/12.]; and IV/10161-1/2020). The data that support the findings of this study are available from the corresponding author on reasonable request.

**Figure.**
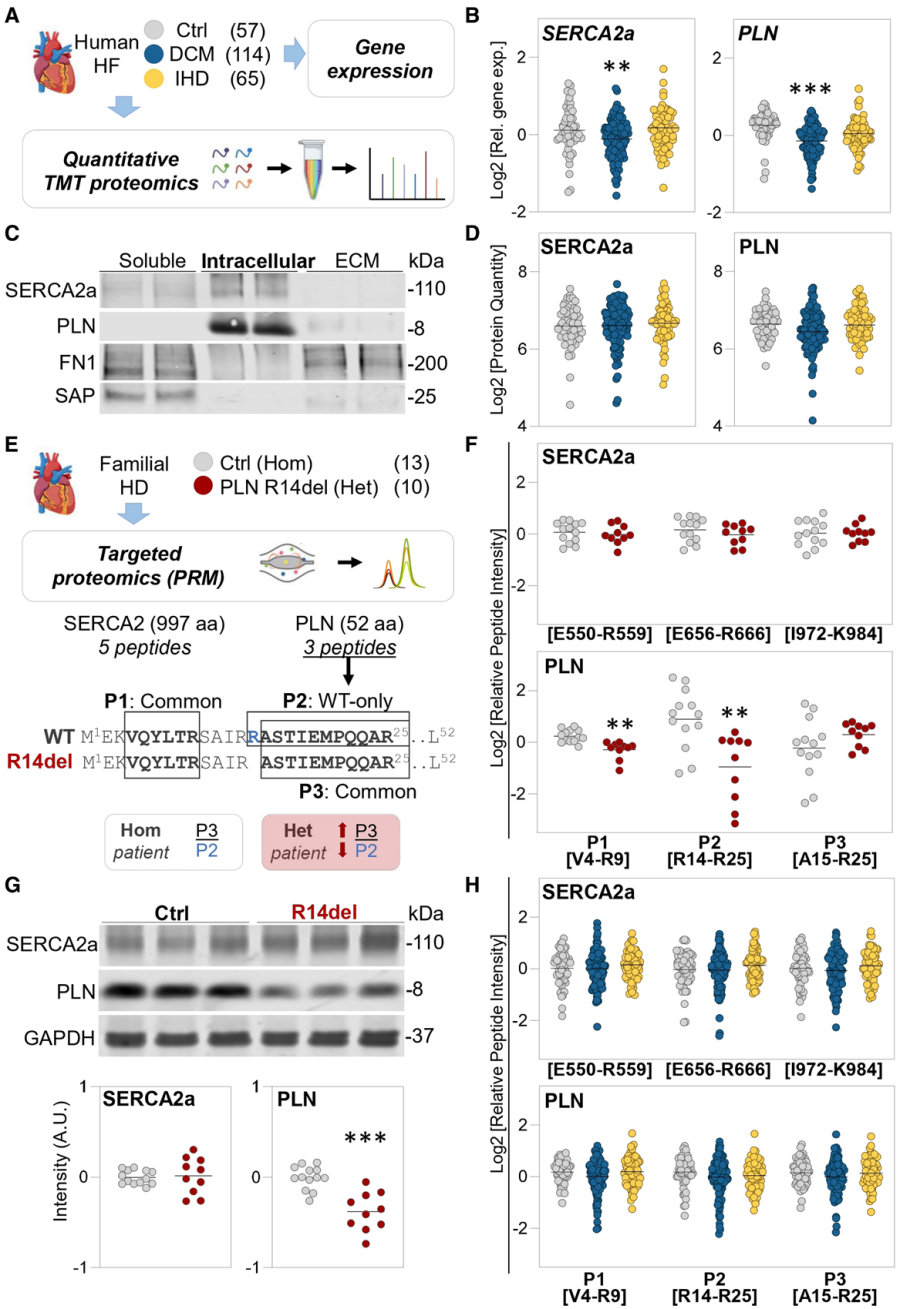
**SERCA2a protein levels in human heart failure. A**, Schematic representation of the workflow used for analyzing cardiac explants from patients with end-stage heart failure (HF) and nonfailing hearts as controls (Ctrl). Gene expression was assessed by reverse transcription–quantitative polymerase chain reaction. The proteomic workflow includes tandem mass tag (TMT) labeling followed by mass spectrometry. **B**, Relative gene expression for sarcoplasmic reticulum Ca^2+^ATPase 2a (SERCA2a) and phospholamban (PLN). Black line represents the mean of the Log2 relative gene expression. Glyceraldehyde 3-phosphate dehydrogenase (GAPDH) was used as normalizer. **P<0.01, ***P<0.001 by Ebayes empirical method of the limma package corrected for age and sex. **C**, Distribution of SERCA2a and PLN after a 3-step extraction process from cardiac tissue. A representative Western blot is shown with 2 pooled samples of the soluble, intracellular, and extracellular matrix (ECM)–enriched protein extracts. **D**, Cardiac protein levels of SERCA2a and PLN as measured by multiplexed TMT proteomics. Black line represents the mean of the Log2 protein levels, normalized and scaled to the sample pool in the TMT reference channel. No significant differences were detected by Ebayes empirical method of the limma package in both uncorrected analysis and after correction for age and sex. **E**, Schematic representation of the targeted proteomics workflow for analyzing hearts from patients with the R14del variation and nonfailing hearts as controls. Parallel reaction monitoring (PRM) was used to quantify 5 proteotypic peptides for SERCA2a and 3 proteotypic peptides for PLN. PLN peptide 1 (P1; V4–R9) and peptide 3 (P3; A15–R25) are common to both the wild-type (WT) and R14del alleles. PLN peptide 2 (P2; A14–R25) is WT specific. Trypsin cleaves PLN at either R13 or R14. The presence of the R14del variation shifts the tryptic cleavage site by 1 amino acid (from P2 to P3 in heterozygous [Het] patients). **F**, Quantification of SERCA2a and PLN by PRM in carriers of the R14del PLN variation and control hearts. Three representative peptide quantifications are shown for SERCA2a, and all 3 targeted peptide quantifications for PLN are displayed. The position of each peptide in the target protein is indicated. Black line represents the mean of the Log2 peptide intensity normalized to the total signal intensity per sample. **P<0.01 by Ebayes empirical method of the limma package and corrected for age and sex. **G**, Representative Western blot and relative quantification of SERCA2a and PLN on cardiac tissue lysates from patients with PLN carrying the R14Del variation and nonfailing hearts as controls. Intensity values are given as arbitrary units (A.U.) after normalization on GAPDH as loading control. ***P<0.001 by Ebayes empirical method of the limma package corrected for age and sex. **H**, SERCA2a and PLN protein levels were measured with PRM in explanted hearts from patients with end-stage HF and nonfailing hearts as controls. Three representative peptide quantifications are shown for SERCA2a. All 3 targeted peptide quantifications for PLN are shown. Each peptide position in the target protein is indicated. Black line represents the mean of the Log2 peptide intensity normalized to the total signal intensity per sample. No significant differences were detected by Ebayes empirical method of the limma package both in uncorrected analysis and after correction for age and sex. aa Indicates amino acid; DCM, dilated cardiomyopathy; FN1, fibronectin; HD, heart disease; Hom, homozygous; IHD, ischemic heart disease; and SAP, serum amyloid P component.

As expected, patients with HF demonstrated increased expression of the precursor for atrial natriuretic factor and a reduced ratio of myosin heavy chain α/β transcripts (data not shown). The transcript levels of the cardiomyocyte-specific isoform *SERCA2a* and *PLN* were lower in cases with DCM compared with controls (Figure [B]). No differences were observed in individuals with IHD. Proteins were extracted following a 3-step methodology to obtain soluble, intracellular, and extracellular matrix proteins.^4^ SERCA2a and PLN proteins were detected in the intracellular fraction (Figure [C]). Therefore, we conducted multiplexed, quantitative proteomics analyses of the intracellular extracts using a tandem mass tag–based workflow. The mass spectrometry spectra for SERCA2 were specifically assigned to the SERCA2a isoform. Although protein levels of atrial natriuretic factor were increased in both patients with DCM and those with IHD compared with controls (fold change, 2.84, *P*<0.001; and fold change, 2.31, *P*=0.019) and inversely correlated to SERCA2a (Spearman Rho=−0.48, *P*<0.001), we detected no significant changes in SERCA2a or PLN protein abundance between DCM, IHD, and control hearts (Figure [D]). Standard-of-care medication was comparable in both HF groups, but patients with IHD had higher use of antiplatelet drugs and statins.

The absence of significant differences in SERCA2a and PLN protein levels led us to investigate their levels in patients heterozygous for the deletion of arginine 14 (R14del) of PLN.^5^ We obtained left ventricles from patients who carry the R14del PLN variation (n=10) from the University Medical Centre Utrecht biobank (UCC-UNRAVEL No. 12-387 and ERN GUARD-Heart, Netherlands) for comparison with nonfailing control hearts (n=13). To quantify the levels of both wild-type and mutant PLN, as well as SERCA2a, we created a targeted proteomics assay using parallel reaction monitoring (Figure [E]). We measured 5 proteotypic peptides for SERCA2a and 3 proteotypic peptides for PLN. PLN peptide 1 (from amino acids V4–R9) is not affected by the R14del variation. However, PLN peptide 2 (from amino acids R14–R25) and PLN peptide 3 (from amino acids A15–R25) are generated by tryptic cleavage at R13 and R14, respectively. PLN peptide 2 is specific to the wild-type allele. In contrast, the generation of PLN peptide 3 will increase with the R14del variation ([Figure E]). PLN peptide 1 was reduced in patients with the R14del variation compared with controls (Figure [F]). The wild-type–specific PLN peptide 2 exhibited a further decrease in patients carrying the R14del variation. On the other hand, PLN peptide 3 showed no reduction due to its increased production in the presence of the R14del variation. We observed no changes in SERCA2a abundance between carriers of the R14del variation and controls (3 representative peptides are shown). The results were further validated by immunoblotting (Figure [G]).

The targeted proteomics assay was also used in our larger cohort of patients with end-stage HF without variations in SERCA2a and PLN. The parallel reaction monitoring approach confirmed that there were no significant differences in SERCA2a and PLN protein levels among patients with DCM, those with IHD, and controls (Figure [H]).

This largest proteomics analysis of cardiac tissues from patients with end-stage HF to date using both untargeted and targeted proteomics showed no differences in SERCA2a and PLN protein abundance compared with control hearts. Our findings challenge the existing paradigm that a reduction of SERCA2a is a hallmark of human HF. Even in carriers of the R14del PLN variation, SERCA2a protein levels were unaltered. Although unaltered protein levels do not imply unaltered SERCA2a activity, our observations suggest that the failure of SERCA2a overexpression in clinical trials may be attributed in part to the absence of a deficiency in SERCA2a protein. The availability of extensive proteomics data sets from human cardiac tissue may guide target selection for future clinical trials.

## ARTICLE INFORMATION

### Sources of Funding

I. Ragone’s PhD project was funded by the European Union’s Horizon 2020 research and innovation program under the Marie Skłodowska-Curie grant agreement 813716. Dr Barallobre-Barreiro is a British Heart Foundation (BHF) Intermediate Fellow (FS/19/33/34328). Dr Mayr is a BHF Chair Holder (CH/16/3/32406) with BHF Programme Grant support (RG/F/21/110053). Drs Theofilatos and Mayr were supported by a BHF project grant (PG/20/10387). Dr Domenech’s work was supported by project PI16/02049 integrated into the National Plan for Scientific Research, Development and Technological Innovation, 2013 to 2016, and funded by the ISCIII–General Subdirection of Assessment and Promotion of Research–European Regional Development Fund. Dr Merkely’s work was funded by the Ministry of Innovation and Technology of Hungary from the National Research, Development and Innovation Fund, financed under the TKP2021-EGA-23 funding scheme. Project RRF-2.3.1-21-2022-00003 has been implemented with the support provided by the European Union. This project was supported by a grant from the National Research, Development and Innovation Office (NKFIH) of Hungary (K134939). Dr Bödör received funding from the Ministry of Innovation and Technology of Hungary from the National Research, Development and Innovation Fund, financed under the TKP2021-EGA-24 and TKP2021-NVA-15 funding schemes. Dr van Veen received financial support from the Netherlands Heart Institute in conjunction with the PLN Genetic Heart Muscle Disease Foundation (CardioVascular Alliance with support of the Dutch Heart Foundation, DCVA2018-30 PREDICT2). Dr Mayr received support from the PLN Genetic Heart Muscle Disease Foundation.

### Disclosures

None.
